# Advances in therapeutic use of a drug-stimulated translational readthrough of premature termination codons

**DOI:** 10.1186/s10020-018-0024-7

**Published:** 2018-05-29

**Authors:** Maciej Dabrowski, Zuzanna Bukowy-Bieryllo, Ewa Zietkiewicz

**Affiliations:** Institute of Human Genetics; Polish Academy of Sciences, Poznan, Poland

**Keywords:** Translational readthrough, Stop codon suppression, Nonsense suppression, Premature termination codon, Genetic diseases

## Abstract

Premature termination codons (PTCs) in the coding regions of mRNA lead to the incorrect termination of translation and generation of non-functional, truncated proteins. Translational readthrough of PTCs induced by pharmaceutical compounds is a promising way of restoring functional protein expression and reducing disease symptoms, without affecting the genome or transcriptome of the patient. While in some cases proven effective, the clinical use of readthrough-inducing compounds is still associated with many risks and difficulties. This review focuses on problems directly associated with compounds used to stimulate PTC readthrough, such as their interactions with the cell and organism, their toxicity and bioavailability (cell permeability; tissue deposition etc.). Various strategies designed to overcome these problems are presented.

## Background

Most of the reviews on the premature termination codons (PTCs) readthrough studies focus on the efficiency of the PTC suppression-stimulating agents, without exploring the associated problems of these agents’ toxicity and bioavailability. The present review, by providing a different perspective, aims to contribute to understanding of the problems associated with using PTC readthrough strategies. The risks and difficulties associated with compounds used to stimulate PTC readthrough, mostly related with their interaction with the cell and organism, and concerning their toxicity and bioavailability (cell permeability; tissue deposition etc.) are discussed. In addition, strategies to overcome these problems are demonstrated. We also present outcomes of recently finished clinical trials on the readthrough compounds. The aspect of PTC identity and its nucleotide context has been recently reviewed (Dabrowski et al., 2015) and is not extensively discussed here; other problems, related to the molecular side of PTC-RT, deserve a separate review and are only briefly mentioned.

## Main text

### Premature termination codons

An extensive meta-analysis study, based on the Human Gene Mutation Database, has revealed that 12% of all described gene lesions causing human inherited diseases is caused by nonsense mutations (Mort et al., 2008). These mutations, by changing an amino acid coding triplet into a stop codon, introduce premature termination codons, PTCs, into the protein-coding gene sequence. PTCs may also be caused by other types of mutations, such as frameshifts (insertion or deletion other than multiple-of-three base pairs) or mutations in the conserved splice-site sequences (leading to a defective intron removal from the pre-mRNA) (Mort et al., 2008; Mendell & Dietz, 2001). PTCs in mRNAs can even occur independently of any changes in DNA, through the aberrant mRNA processing (alternative splicing) (Pan et al., 2006; Lewis et al., 2003).

In any case, PTCs located in the coding regions of mRNA lead to the premature termination of translation and, as a consequence, to the production of truncated proteins (Mendell & Dietz, 2001). In most cases, this reduces the amount of a full-length protein in a recessive-negative manner, whereby mutations of both alleles are necessary to result in a deleterious phenotype (Khajavi et al., 2006). However, PTCs can also exert dominant-negative effect (Inoue et al., 2004). Phenotypic manifestation usually involves loss-of-function effects, but there are examples of gain-of-function events (Manuvakhova et al., 2000). For example, in β-thalassemia, truncated proteins derived from PTC-bearing transcripts are responsible for generation of insoluble globin chains, which are toxic to the cell (Thein et al., 1990).

The synthesis of deleterious C-terminally truncated proteins in eukaryotic organisms is reduced due to the specialized mRNA surveillance mechanisms. One of them is the nonsense-mediated mRNA decay (NMD) pathway **(**Lejeune, 2017a**)**. This conserved quality-control process detects and degrades transcripts containing abnormalities such as: PTCs, introns downstream of the termination codon, long 3′ untranslated regions (3′UTRs) or upstream open reading frames (uORFs) (Mühlemann, 2008; Hogg & Goff, 2010; Miller & Pearce, 2014; Lejeune, 2017b).

Many efforts have been made to develop therapeutic strategies that would counteract negative effects of PTCs. One of them relies on exploring the natural phenomenon of termination codon suppression through the translational readthrough mechanism.

### Basal translational readthrough

Effective termination of protein translation in eukaryotes requires recognition of the stop codon, localized in the A-site of the ribosome, by a release factor, eRF1. The eRF1 further interacts with another release factor – eRF3, and with GTP. This ternary termination complex (eRF1-eRF3-GTP) interacts with the poly(A)-binding protein (PABP) present at the 3′-UTR of mRNA; this leads to efficient hydrolysis of GTP and cleavage of the peptidyl-tRNA bond (Bulygin et al., 2017). As a consequence, the newly synthesized polypeptide chain is released from the ribosome.

**Fig. 1:**
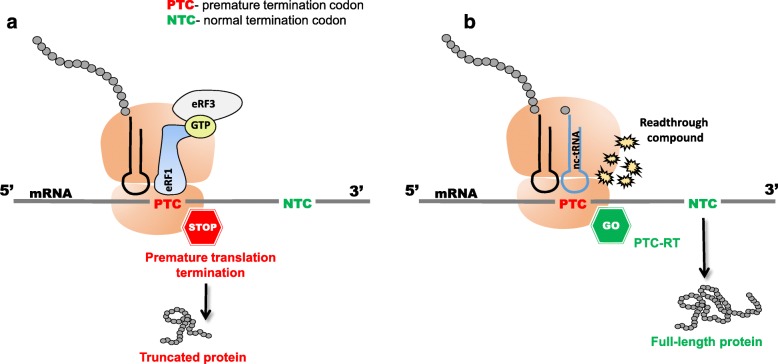
PTC-RT process. **a** Ribosome encounters premature termination codon (PTC); the site is recognized by the translation termination machinery and the polypeptide elongation is prematurely terminated. **b** After addition of readthrough compound, translational machinery decodes PTC (PTC is recognized by nc- tRNAs), and translation continues until the normal termination codon (NTC). It allows translating a full-length protein. The NMD surveillance mechanism may detect and degrade PTC-bearing transcripts. If the NMD process is inhibited, very low levels of the full-length protein can be present even in the absence of stimulating agents, as the result of the endogeneous suppression of PTC

With the termination of translation being based on the steric match between the stop codon and the eRF1, this process is not 100% efficient. Stop codons in mRNA can be suppressed (recoded) through the natural mechanism of basal translational readthrough (Dabrowski et al., 2015; Fearon et al., 1994). In this process, a near-cognate tRNA (nc-tRNA) outcompetes eRF1 at the A-site of the ribosome. Nc-tRNAs have anticodons, which are complementary to only two of the three positions of a nonsense codon in mRNA. The recent studies indicate, that the interaction between PTC and a nc-tRNA anticodon occurs by mispairing at either position 3 or 1 of the stop codon (Roy et al., 2015; Roy et al., 2016). If the nc-tRNA interacts with the PTC at the A-site, the amino acid transported by such nc-tRNA is incorporated into the synthesized polypeptide chain, and translation – instead of being terminated – continues until the next in-frame stop codon (Fig. 1).

Not all stop codons can be read through with the same efficacy. The level of “leakiness” of the stop codons can be ranked as UGA > UAG > UAA (Manuvakhova et al., 2000). Also the sequence of nucleotides upstream and downstream to the stop codon has the effect on the efficacy of the readthrough process. The majority of evidence indicates that a nucleotide immediately following the termination codon in the 3′ direction (position + 4, when the first nucleotide of the stop codon is marked as + 1), is involved in the interactions between mRNA and translational machinery (McCaughan et al., 1995; Jungreis et al., 2011; Loughran et al., 2014). This suggests that the actual translation termination signal consists of a tetranucleotide sequence, rather than only the stop codon itself (Brown et al., 1990). Recently, it has been proposed, based on genetic interaction studies in yeast, that + 4 cytosine compromises the ability of eRF1 to recognize appropriately a stop codon, thus providing precedence for the importance of nucleotide positioning for eRF1-mediated termination efficiency (Beznosková et al., 2016). In addition, the upstream sequence, immediately 5′ of the stop codon (positions − 1 to − 3) also exerts an effect; this effect is considered to be more subtle than the downstream sequence context of the transcript. Studies suggest that even relatively distant nucleotides (positions + 5, + 6, + 9) may also influence the translational readthrough (for a review, see Dabrowski et al., 2015, RNA Biology) (Dabrowski et al., 2015).

It has been estimated that basal readthrough of normal termination codons (NTCs) occurs in 0.001 to 0.1% of total rounds of translation of a given transcript (Keeling et al., 2012). In the case of PTCs, basal readthrough levels are higher and can range from 0.01% to even 1% (Manuvakhova et al., 2000; Bonetti et al., 1995; Cassan & Rousset, 2001). It is not known, why the basal readthrough levels differ so much. It has been hypothesized, that efficient translation termination requires the close contact between the release factors interacting with NTC and PABPs (Lejeune, 2017a; Cosson et al., 2002; Celik et al., 2015). Presence of a PTC in the transcript, usually more distant from the 3’UTR, could limit the interaction between eRFs and PABPs, leading to the less efficient action of eRF3 and the delayed release of the transcript from the ribosome (Ivanov et al., 2008). The prolonged presence of the translational machinery at the PTC would increase the susceptibility to the basal translational readthrough of the PTC (PTC-RT) (Amrani et al., 2004).

### Drug-induced translational readthrough

**Fig. 2:**
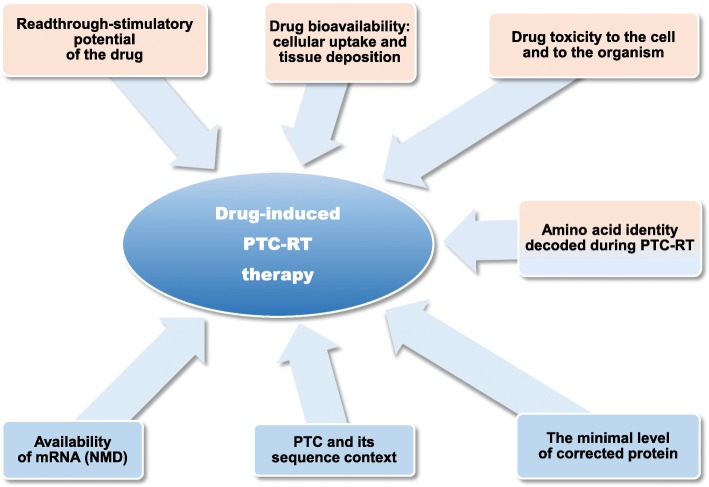
Different problems associated with the drug-stimulated PTC-RT therapy. Upper part – problems associated with the PTC-RT- stimulating drugs. Lower part – problems associated with the biology of PTC-RT process

A number of studies have demonstrated that certain low-molecular-mass drugs can stimulate recoding of a PTC by the translation machinery (Lee & Dougherty, 2012). These findings have opened the way to new therapeutic approaches to nonsense mutations in genes associated with genetic diseases. However, the efficient use of this approach in the clinic is still hampered by a number of problems (Fig. 2).

Aminoglycoside antibiotics (AAGs) are among the most studied drugs capable of inducing PTC-RT - already in 1960s, first papers indicating readthrough potential of AAGs in bacteria have been published (Davies et al., 1964; Anderson et al., 1965). In 1985, this process was demonstrated in mammalian cells (Burke & Mogg, 1985). AAGs are oligosaccharides consisting of streptidine or 2-deoxystreptidine as the molecular core, with a variable number of sugar rings and ammonium groups (François et al., 2005). Commonly used to treat Gram-negative bacterial infections, they bind with the seven-nucleotide loop structure in the decoding center of the bacterial ribosome and interfere with its function (Fan-Minogue & Bedwell, 2008). This enables effective misincorporation of the nc-tRNAs, resulting in extensive translational misreading, followed by a complete inhibition of protein synthesis in bacteria. In eukaryotes, a small difference in the rRNA nucleotide sequence significantly lowers the efficiency of this interaction (Lynch & Puglisi, 2001). Nonetheless, in the case of PTC, the impact of AAGs on the translational apparatus is often sufficient to reduce discrimination between cognate and nc-tRNAs, and to enhance translational readthrough.

The therapeutic potential of AAGs as PTC readthrough-stimulating compounds has been tested in many models, from the in vitro transcription and translation system (Manuvakhova et al., 2000; Du et al., 2002; Keeling & Bedwell, 2002; Lai et al., 2004), through dual reporters in cell lines (Howard et al., 2004; Bidou et al., 2004; Floquet et al., 2011; Bukowy-Bieryllo et al., 2016), patient derived cells (Salvatori et al., 2009; Nakamura et al., 2012; Cogan et al., 2014; Dündar et al., 2017). The first in vivo demonstration of the therapeutic AAG properties has been in mdx mice, an animal model of Duchenne muscular dystrophy (DMD), where gentamicin treatment restored 10–20% of normal dystrophin levels in the skeletal muscles (Barton-Davis et al., 1999). Since then, a large variety of AAGs has been tested for their PTC-RT-stimulating properties, both in animal models (Arakawa et al., 2003; Du et al., 2006; Guerin et al., 2008; Yukihara et al., 2011; Rowe et al., 2011; Gunn et al., 2014) and clinical trials (Clancy et al., 2001; Wilschanski et al., 2003; Politano et al., 2003; Sermet-Gaudelus et al., 2007; Malik et al., 2010). The latest pilot clinical study (ClinicalTrials.gov; id: NCT02698735) has shown positive results of gentamicin use in recessive dystrophic epidermolysis bullosa patients with nonsense mutation in the gene encoding type VII collagen. Topical or intradermal administration of gentamicin increased the level of full-length collagen VII from 20 to 165% of the wild-type level, the expression of the protein persisted for 3 months. In addition, a significant reduction of disease symptoms was observed (improved epidermal-dermal adherence, reduced blister formation, and enhanced wound closure) (Woodley et al., 2017).

#### Toxicity of AAGs

Even though AAGs have been commonly used in clinics for decades, they may exert strong oto- and nephrotoxic effects on the organism. Accumulation of AAGs in the renal epithelial cells leads to apoptosis and necrosis of these cells (Lopez-Novoa et al., 2011). However, this effect is reversible after stopping AAGs administration (Mingeot-Leclercq et al., 1999). The negative influence of AAGs on the renal function in patients can be also diminished by the appropriate hydration therapy and application of dialysis, when necessary (Lopez-Novoa et al., 2011). The ototoxicity induced by AAGs manifests as irreversible, symmetric, bilateral sensorineural hearing loss. Due to the very slow removal of AAGs from the inner ear fluids, the onset of hearing loss can occur days, or even weeks, after finishing the AAG administration (Huth et al., 2011). Inside the hair cell, AAGs cause damage, either directly or indirectly, first by causing disarray of stereocilia, and ultimately by activating apoptosis (Abi-Hachem et al., 2010).

The selective toxicity observed in the inner ear and kidneys is associated with the cellular uptake of AAGs through megalin, an endocytic receptor localized in the apical membrane of epithelial cells present in the proximal renal tubules and in the hair cells of the inner ear (Moestrup et al., 1995). The mechanism, by which AAGs induce apoptosis, both in the renal cells and in the hair cells, is to date not well-defined. Some evidence indicates a role of reactive oxygen species (ROS) (Huth et al., 2011). Positively-charged AAGs are able to interact with various negatively-charged cellular components such as phospholipids, phospholipases, and metal ions. AAGs bounded to phospholipids form complexes, which can aggregate in the internal lysosomal membranes, causing phospholipidosis, a condition often associated with nephro- and ototoxicity (Couture et al., 1994; Reasor & Kacew, 2001). More importantly, the AAG-phospholipid interaction leads to the generation of ROS, which affect membrane fluidity and permeability, disrupt the activity of enzymes, ion channels and receptors, and finally direct the cell to the apoptotic pathway (Xie et al., 2011). Another explanation for the cell apoptosis related to AAGs administration suggests involvement of the decoding site of the ribosome of mitochondrial ribosomes, which closely resembles the A-site of bacterial ribosomes. The interaction of AAGs with the 12S rRNA at the A-site causes mistranslation, then inhibition of protein synthesis, ultimately leading to cell death (Hobbie et al., 2008). AAG-mediated oxidative damage of the mitochondrial enzyme – aconitase can also cause an increase in superoxide overproduction from the free ferrous iron in mitochondria, that in turn increases the amount of hydroxyl radicals via the Fenton reaction, and finally induces the cell apoptosis (Shulman et al., 2014).

In spite of these adverse effects, one study has demonstrated that AAGs used to stimulate PTC-RT can be relatively safe. The six month-long gentamicin therapy in DMD patients was completed safely, with no impairment of either renal or hearing function. The negative effects of AAGs were avoided thanks to the informed choice of the study cohort and a careful study design; all subjects with the A1555G mutation in 12S rRNA gene of mtDNA, known to predispose to gentamicin-induced ototoxicity, had been excluded from the treatment, while in the remaining patients a strict regimen of gentamicin administration was obeyed (Malik et al., 2010).

Although the clinical use of AAGs for a prolonged time is associated with many risks, the benefits of a possible PTC-RT-based therapy are indisputable. To alleviate the problems resulting from AAGs toxicity, various attempts have been undertaken to mitigate the toxicity or to select other compounds with PTC-RT-inducing potential but without undesirable side effects of AAGs.

#### Overcoming cytotoxic effects of AAGs

One approach tested for reducing the cytotoxicity of AAGs involves their co-administration with different compounds, which are able to reduce interaction of AAGs with different cellular targets. This strategy was shown to be successful for tobramycin or gentamicin co-administrated with a lipopeptic antibiotic, daptomycin (Beauchamp et al., 1990; Thibault et al., 1994). Negatively charged daptomycin complexed AAGs through an electrostatic interaction, preventing them from binding to phospholipids and other intracellular targets (Couture et al., 1994). Co-administration of gentamicin with another compound, the negatively charged poly-L-aspartic acid (PAA), was shown to protect rats against the development of kidney nephrotoxicity (Gilbert et al., 1989; Ramsammy et al., 1989). It has been suggested that PAA, similarly to daptomycin, binds AAGs and prevents phospholipidosis, which would otherwise lead to nephrotoxicity (Kishore et al., 1990). In addition, PAA increased the intracellular concentration of gentamicin, contributing to a higher level of PTC-RT. It also slowed-down elimination of AAGs from cytosol, extending the time period during which readthrough stimulation occurred. Similar results were obtained in the mouse model of cystic fibrosis (Du et al., 2009a).

Another approach used to mitigate the toxic effects of AAGs relies on their co-administration with antioxidants. D-methionine and melatonin were shown to reduce the harmful ROS formation caused by the interaction of AAGs with the cell components (Campbell et al., 2007; Reiter et al., 2011). Melatonin turned out to be particularly effective; compared to a mixture of other antioxidants, it was 150 times more effective in reducing ototoxic effects of gentamicin or tobramycin.

Recently, a new approach to reduce AAG toxicity has been tested, whereby AAGs were co-administered with small molecular mass molecules, derivatives of phthalimide (e.g. CDX5–1) (Baradaran-Heravi et al., 2016). When used alone, these compounds did not induce PTC-RT, but applied with AAGs, they efficiently potentiated PTC-RT stimulating efficacy of AAGs. This strategy allowed significant lowering of the therapeutic dose of AAGs, thus reducing the AAG-related toxicity. For example, CDX5–1 administered together with G418 not only increased the level of PTC-RT up to 180-fold, but also accelerated synthesis of the full-length protein (the full-length product was detected 10 times faster). CDX5–1 action did not rely on stimulating PTC-RT; nor did it inhibit the NMD process (Baradaran-Heravi et al., 2016). The mechanism by which this compound potentiates PTC-RT activity of AAGs, as well as its potential interference with other cellular processes, remains to be elucidated.

Other approaches aiming to reduce AAG toxicity related to their positive electrostatic charge involve the use of liposome-encapsulated AAGs. Liposomes containing encapsulated gentamicin, used to stimulate PTC-RT in DMD mice, were more effective and caused 10-fold lower ototoxicity than the traditionally administered gentamicin. Creatinine concentrations (an indicator of renal malfunction) in mice treated with these compounds were normal, suggesting that encapsulation of AAG also reduced nephrotoxicity (Yukihara et al., 2011; Schiffelers et al., 2001).

#### Chemical modification of AAGs - AAG derivatives

A different approach to the therapeutic use of PTC-RT involves modification of the chemical structure of AAGs with a proven nonsense suppressing ability, to obtain compounds with a reduced toxicity and retained or increased therapeutic potential. An example of this approach comes from the studies on artificially designed paromomycin derivatives, where NB30 represents the first, and NB54 – the second generation. The modification of the paromomycin structure effectively reduced toxicity of the derivative compounds. For example, NB30 was 15-fold less toxic than gentamicin, while both compounds showed nonsense suppression at the levels similar to those induced by gentamicin and paromomycin (Nudelman et al., 2009). The compounds were able to induce statistically significant PTC-RT levels in cell lines from patients with different PTC-caused diseases, such as: Rett syndrome (Brendel et al., 2011; Vecsler et al., 2011), cystic fibrosis (Rowe et al., 2011), mucopolysaccharidosis type I – Hurler syndrome (MPS I-H) (Wang et al., 2012; Kamei et al., 2013) and Usher syndrome type 1 (Rebibo-Sabbah et al., 2007; Goldmann et al., 2012).

Modification of the structure of another AAG, G418, resulted in the third generation of PTC-RT-stimulating AAG derivatives, NB74 and NB84. These compounds were several-fold more active than the previous generations (Nudelman et al., 2010). Importantly, modifications also highly reduced the toxicity of the compounds. The capacity of NB74 and NB84 to stimulate PTC-RT was analyzed in the mouse model of MPS I-H caused by a PTC mutation in the α-L-iduronidase gene. The level of PTC-RT induced by NB84 was higher than that caused by gentamicin or by the first and second generation of AAG derivatives, and the LC50 values were 9–10 times higher than for G418 (Shulman et al., 2014). A long-term (28-weeks) treatment of MPS I-H mice with NB84 resulted in an increased activity of α-L-iduronidase in different tissues, including the brain, heart and bone. The level of the enzyme was sufficient to significantly reduce disease symptoms in the tissues, while no visible toxicity was observed; moreover both the NB84 and NB74 compounds were shown to be able to cross the blood-brain barrier (Gunn et al., 2014). The newest representative of the third generation of chemically modified AAGs, NB124, turned out to be even more promising. Its therapeutic index (comparison of the amount of a compound that causes the therapeutic effect to the amount that causes toxicity) was 10 times better than that obtained for gentamicin and other non-modified AAGs. Moreover, a series of cell and animal based experiments using the CF models have shown that NB124 restored up to 10% of the wild-type CFTR function (Xue et al., 2014). In the most recent study, using reporter vectors in the transiently transfected cell lines, NB124 was shown to efficiently decode common PTC mutations in tumor suppressor genes (p53 and APC) in the murine cell line (NIH3T3), resulting in over 20% of the PTC-RT. In the HDQ-P1 cell line (derived from human primary breast carcinoma), the restoration of p53 and APC tumor suppressor proteins expression by NB124 induced apoptosis in 38% of the cells, whereas untreated cells displayed no signs of apoptosis. In addition, similarly to G418, NB124 also affected the NMD process, inhibiting the degradation of p53 transcripts (Bidou et al., 2017).

Another modified AAG is a pyranmycin (TC007), a derivative of another aminoglycoside antibiotic, neomycin (Chang et al., 2002). In the fibroblasts from patients with spinal muscular atrophy (SMA) caused by the premature termination of the SMN protein, stimulation with TC007 resulted in the 10-fold increase in the level of full-length SMN protein. In a mouse model of SMA, injection of TC007 into the central nervous system (brain ventricles) induced higher expression SMN protein, led to a longer survival of the motor neurons and was associated with a 27% increase in the lifespan of the SMA mice (Mattis et al., 2009; Mattis et al., 2012).

#### Readthrough compounds

Apart from modification of AAG structure, a number of studies have searched for readthrough-inducing compounds, chemically not related to AAG, but possessing the potential to suppress nonsense mutations in mammalian cells (Lee & Dougherty, 2012). The best known example of this group is a small molecule drug 3-[5-(2-fluorophenyl)-1,2,4-oxadiazol-3-yl]-benzoic acid, also known as PTC124, Ataluren or Translarna. Identified during a high-throughput screen, PTC124 has been selected as the most potent readthrough promoting drug from over 800,000 compounds screened (Welch et al., 2007).

PTC-RT stimulating efficiency of PTC124 was successfully demonstrated in animals (Goldmann et al., 2012; Welch et al., 2007). It was also tested in different in vitro and ex vivo models of PTC-mediated diseases, including cystic fibrosis (Du et al., 2008), Duchenne muscular dystrophy (Yukihara et al., 2011; Finkel et al., 2013), Miyoshi myopathy (Wang et al., 2010), Usher syndrome (Goldmann et al., 2012) and Batten disease (Sarkar et al., 2011). Phase I and phase II clinical trials have shown that its adverse effects were mild or moderate and similar to the placebo-treated group; therefore, PTC124 has been deemed safe for therapeutic use in humans (Hirawat et al., 2007; Kerem et al., 2008), and a number of clinical trials have been undertaken. Unfortunately, despite many positive results from different experimental models, the effects of PTC124 in patients remain inconclusive.

In CF patients with PTC mutations in the *CFTR* gene, treatment with PTC124 slightly increased chloride channel activity, resulting in an improvement of clinical parameters (Kerem et al., 2008); however, not all patients responded positively to the drug. In a similar clinical trial with pediatric CF patients, an improvement in chloride channel activity was observed again, and the presence of CFTR protein in nasal epithelium was confirmed by immunofluorescence; nevertheless, no significant therapeutic improvement was proved (Sermet-Gaudelus et al., 2010). In the phase III clinical trials, some improvement in lung function and reduction in exacerbation frequency were reported after 48 weeks of PTC124 therapy, although statistical significance in the overall patient population was not reached (Kerem et al., 2014). The main outcome of another phase III clinical study (ClinicalTrials.gov; id: NCT02139306, finished in March 2017), has been announced via the PTC Therapeutics press release, and at the time of writing this review, the full data were still unpublished. The announcement reported a failure to reach the expected primary and secondary endpoints. In light of these discouraging results, PTC Therapeutics has recently decided to discontinue their clinical development of PTC124 for CF and withdraw its application for marketing authorization in Europe (PR Newswire).

PTC124-induced suppression of PTC was also tested in clinical trials with DMD patients. In the phase IIA trial, an increased expression of the full-length dystrophin was shown in patients’ biopsies after 28 days of oral administration of PTC124; one third of the patients demonstrated an increase in the post-treatment expression of dystrophin appropriately localized in the sarcolemmal membrane of muscle cells (Finkel et al., 2013). In the phase IIb trial, the patients showed a slower decline in the walk test; no improvement related to the higher dose of PTC124 was observed, suggesting a bell-shaped dose-response curve (Bushby et al., 2014). Unfortunately, phase III clinical study completed in 2017, indicated that similarly to the CF studies, the effects of PTC124 treatment did not differ significantly from the placebo group (McDonald et al., 2017).

Despite the intensive studies, the exact mechanism of PTC124 action remains unknown. Computational modeling suggested that mRNAs containing PTC form stable complexes with PTC124, which may interfere with the recognition of PTC by eRF1 and suppress the termination of translation; however, this interaction was shown to be stable only for the UGA codon (Lentini et al., 2014). In cell lines transfected with different PTC-bearing constructs, the PTC-RT-stimulating potential of PTC124 was different for distinct stop codons; UGA suppression was three times more effective than UAG, and 6 times more effective than suppression of UAA (Welch et al., 2007). The latest evidence indicates, that PTC124 has a selectivity for the ribosomal A site and that it promotes insertion of nc-tRNAs at the PTC site (Roy et al., 2016). Tobramycin, a compound with an affinity for the ribosomal A site, is a strong inhibitor of PTC124 (probably by competition), what supports the expected interaction of PTC124 with the ribosome (Roy et al., 2016; Salian et al., 2012). Moreover, in a post-hoc analysis of the recent clinical trial, it was observed that CF patients with nonsense mutations failed to show a significant response to administered PTC124, if they were concurrently treated with the inhaled tobramycin (Kerem et al., 2014).

Clitocine [6-amino-5-nitro-4-(β-D-ribofuranosylamino)pyrimidine] has been identified in the same high-throughput screen as PTC124 (Welch et al., 2000). This compound is an adenosine nucleoside analog originally isolated from the mushroom *Clitocybe inverse* (Kubo et al., 1986). Clitocine is incorporated into mRNA during transcription as an adenosine substitute; unlike AAGs that act on the translation machinery, clitocine facilitates decoding of the nonsense codon just by its presence in the transcript (Friesen et al., 2017). Clitocine can be incorporated instead of the adenine in each of the stop codons. The order of readthrough susceptibility of the stop codons in the presence of clitocine is UAA> > UGA > UAG. Surprisingly, the UAA codon is the most leaky, probably because of the incorporation of clitocine in both the second and third position of the codon (Friesen et al., 2017). Stop codons with the incorporated clitocine are poorly recognized by the eRF1 translation termination factor; this prevents efficient termination, enhances nc-tRNA recruitment and therefore increases the level of PTC-RT. In the mentioned studies, a full-lengh p53 protein that was translated due to the readthrough was fully functional, both in the cell lines with PTC-containing alleles and in the tumor-bearing mice (Friesen et al., 2017).

Apart from PTC124, a number of other low-molecular-mass compounds, capable of stimulating PTC-RT, have been identified to date. Screening of ~ 34,000 compounds using a novel high-throughput screening assay identified twelve most promising readthrough-stimulating compounds (Du et al., 2009b). The compounds displayed reduced toxicity in the mammalian cells and did not alter the global protein expression patterns (Gatti, 2012). Two leading first generation compounds, RTC13 and RTC14, were proven to suppress nonsense mutations in ataxia telangiectasia patients’ cell lines (A-T cells) and in myotube cells from *mdx* mice (Du et al., 2009b; Kayali et al., 2012). More recent studies identified the further PTC-RT-stimulating compounds, namely GJ071 and GJ072, their analogs (RTC204 and RTC219), as well as non-related compounds, BZ6 and BZ16 (Jung et al., 2011; Du et al., 2013). In the A-T cells, these compounds displayed PTC-RT-stimulating efficiency comparable to RTC13, RTC14 and PTC124, but with significantly lower toxicity. When tested in cell lines from patients with four different lysosomal storage diseases, RTC13, RTC14, BZ6 and BZ16 showed ~ 1.5 fold increase in the amount of a relevant full-length mRNA compared to the previously used compounds (Gómez-Grau et al., 2015) and were able to suppress all three stop codons (Du et al., 2013). However, the mechanism of their action remains unknown.

New readthrough compounds have also been found among non-AAG antibiotics. A dipeptide antibiotic, negamycin, structurally not related to AAG, affects the process of ribosomal decoding in a way similar to AAGs (Arakawa et al., 2003). However, in contrast to AAG, negamycin exhibits much lower cytotoxicity and no ototoxicity. Negamycin was shown to promote PTC-RT in the dystrophin gene both in vivo in the mouse model of DMD and in the cultured *mdx* myotubes (Arakawa et al., 2003). It was also tested in congenital muscular dystrophy: PTC-RT induced by negamycin in the transfected NIH3T3 cells was several-fold higher than that induced by AAGs (Allamand et al., 2008).

Another group of compounds capable of inducing PTC-RT are macrolides, antibiotics often used to treat Gram-positive bacterial infections. Induction of PTC-RT by tylosin, josamycin, and spiramycin was demonstrated in colorectal cancer models (mutations in the *APC* gene) (Zilberberg et al., 2010). The effect was also confirmed in a xenograft mouse model – treatment with macrolides resulted in reduced tumor growth and led to a notable increase in the lifespan of the mice (Zilberberg et al., 2010). These data were later extended by the flow cytometry-based reporter assay, in which erythromycin and its derivative, azithromycin, were used (Caspi et al., 2015). Macrolides were analyzed in cell lines derived from patients with mutations in a number of genes; *ATM* in ataxia-telangiectasia (A-T), *MeCP2* in RTT syndrome, *SMN* in the SMA syndrome, *APC* in familial adenomatous polyposis (FAP). In all these cases, macrolides induced PTC-RT; additionally, azithromycin worked at a 100-fold lower concentration than any other compound tested so far, enabling a significant reduction of the therapeutic dose (Caspi et al., 2015). PTC-RT-stimulating potential of azithromycin was also confirmed in SMA mouse model after intracerebroventricular administration of this drug (Osman & Iii, 2017). The mechanism of macrolide action in PTC-RT differs from that of AAGs: according to studies on bacteria, macrolides are thought to influence the mechanism of protein synthesis termination, either by reducing binding of the RF1 or RF2 to the A-site of the ribosome, or by inhibiting the release of a peptide from the ribosome after binding of the release factors is completed (Thompson et al., 2004).

Identification of novel readthrough compounds among antibiotics has inspired the search for readthrough-stimulating properties among other clinically approved drugs, which already have an existing safety profile. A screen of 1600 clinically approved drugs identified an herbal anti-inflammatory drug, escin (Mutyam et al., 2016). Escin induced a significant PTC-RT, both in the in vitro tests and in the cell lines from a CF patient. It also stabilized transcripts by inhibiting NMD, which resulted in a significant increase of the functional protein level in the cells. No side effects have been reported so far, and escin seems to be a promising PTC-RT-stimulating compound. However, further functional tests need to be performed before it can be used in clinical trials.

### Readthrough induced by the use of suppressor tRNAs

There is an alternative for the stop suppression stimulated by readthrough compounds - the suppressor tRNAs. Suppressor tRNAs are altered aminoacylated-tRNAs with anticodons complementary to stop codons (Beier & Grimm, 2001). They do not stimulate translational machinery like others readthrough compounds, but they outcompete translation termination factors (like nc-tRNAs), and drive incorporation of an amino acid, being therefore readthrough agents per se. Suppressor tRNAs have been shown to restore the expression of functional proteins in PTC-carrying human cell models of beta-thalassemia (Temple et al., 1982), xeroderma pigmentosum (Panchal et al., 1999), Ulrich disease (Sako et al., 2006) and, more recently, cancer (Bordeira-Carriço et al., 2014). The PTC suppression using suppressor tRNA was also shown in mice, albeit with very low efficiency (Buvoli et al., 2000). In contrast to the readthrough compounds, which stimulate PTC recognition by nc-tRNAs (resulting in the incorporation of amino acid that does not necessarily correspond to the wild-type), suppressor tRNAs do not introduce missense mutations, and thus lead to the synthesis of the functional protein (Bordeira-Carriço et al., 2014). However, a clinical use of suppressor tRNAs faces many challenges, especially regarding the efficiency of delivery and in vivo expression of these systems, with minimal toxicity to the organism.

### Bioavailability of the drugs used to stimulate PTC-RT

When drug-stimulated PTC-RT is considered in the context of its possible therapeutic application, bioavailability of the readthrough compounds, both at the level of the cell as that of the organism, is an important issue.

### The cell membrane permeability to readthrough compounds

PTC-RT may be limited by the permeability of the cells to readthrough compounds. Uptake of the drugs, especially of AAGs, has been explored for many years, but still no unequivocal conclusions have been reached. Even less is known about the mechanisms of the cell penetrance by the AAG derivatives or non-AAG compounds with PTC-RT-stimulating potential.

Nearly all mammalian cells take up AAGs; these drugs may cross cellular membranes via endocytosis or non-endocytotic pathways (Steyger, 2005). One of the best studied AAGs uptake mechanism is through the multi-ligand endocytic receptor – megalin. Megalin has been localized in the apical membrane of the epithelial cells of the proximal renal tubules and in the hair cells of the inner ear, and is related to the nephro- and ototoxic effects of AAGs (Tauris et al., 2009; Christensen et al., 2012). However, at low temperature, when endocytosis is significantly slowed down, the presence of Texas Red-tagged gentamicin (GTTR) in the cytoplasm or the gentamicin-related ROS production occurred within seconds (Myrdal et al., 2005; Hirose et al., 1997). This suggests that AAGs uptake involves different mechanisms than endocytosis alone.

A potential non-endocytic route of AAGs uptake is through non-selective cation channels (NSCCs), such as TRP channels (Marcotti et al., 2005; Lee et al., 2013; Stepanyan et al., 2011). Low Ca2+ level in the extracellular fluid, hyperpolarization of the cell membrane or application of NTSCC regulators (e.g. TRP agonists: resiniferatoxin and anandamide) activate NSCCs opening, induce influx of cations along with the influx of positively charged compounds. Factors inducing activation of NSCCs opening also favor AAGs uptake (Steyger, 2005). Similar to megalin, TRP channels are present in the membrane of kidney epithelial cells and of inner ear sensory cells, thus may also be involved in oto- and nephrotoxic effects of AAGs. Moreover, AAGs uptake may also be stimulated by loop diuretics, like bumetanide and furosemide. These drugs, which hyperpolarize the cell membrane, were shown to increase the influx of a cationic AAG (GTTR) through NSCCs, enhancing GTTR uptake by 60% (Wang et al., 2013).

### Effective dose of readthrough compounds

Regarding the modes of administration, the choice of doses and duration of therapy is important. The stimulation of PTC-RT is a transient phenomenon, and the therapeutic use of drugs requires a repetitive, prolonged, usually life-long administration of the compounds. Most studies in animal models indicate that the action of AAGs follows a peak-driven mode. For example, in the *mdx* mice, maximal levels of intravenously injected gentamicin in serum were observed 20 min after administration; after 4 h, the drug level rapidly decreased. They also showed that a constant pump-driven administration of low-concentrations of gentamicin did not result in readthrough (Barton-Davis et al., 1999). It was confirmed in *retinitis pigmentosa* mice, that a better response to the treatment was obtained with repeated single injections of a higher dose than with continuous delivery of small doses of AAGs (Guerin et al., 2008). In the clinical trials with CF patients, different regimens of the administered gentamicin were used (Clancy et al., 2001; Sermet-Gaudelus et al., 2007; Malik et al., 2010). In the Clancy’s study, the scheme of intravenous administration of gentamicin (2.5 mg/kg three times a day) was adjusted to achieve peak concentration in the serum from 8 to 10 μg/ml (Clancy et al., 2001). In Sermet-Gaudelus study, gentamicin was administered at 10 mg/kg once daily for 15 days; gentamicin peak concentration was achieved 30 min after the infusion (Sermet-Gaudelus et al., 2007). In another long term study, gentamicin was administered intravenously (7.5 mg/kg) once or twice per week for 6 months; gentamicin efficacy was limited and its toxicity precluded administration of higher doses of this drug (Malik et al., 2010).

Effective dose of the best studied non-aminoglycoside compound, PTC124 was shown on healthy volunteers and CF patients in phase I and II clinical trials. PTC124 was characterized by rapid oral absorption and dose-proportional increases in pharmacokinetic parameters. Peak concentration of this compound was achieved at approximately 2 h after dosing and its half-life ranged between 3 to 6 h (Hirawat et al., 2007). Similarly to AAGs, the best response for the treatment was observed after a multi-dose administration, however without toxic drug accumulation or metabolic auto-induction. The dosage used in clinical trials varied from 16 mg/kg/day in a 14-day treatment period for the first studies to the 40 mg/kg/day in three divided doses for 48 weeks (Kerem et al., 2008; Sermet-Gaudelus et al., 2010). Generally, choosing the most effective regimen of drug administration for a given disease remains challenging.

### Other factors influencing therapeutic potential of drug-induced PTC-RT

The efficiency of PTC-RT stimulation, the toxicity and the bioavailability of stimulating compounds are important issues to be addressed. As presented above, these problems are potentially manageable, mainly through the search for new, more efficient and less toxic compounds, for the means to enhance cellular uptake of the drugs and for the efficient ways to deliver these drug to proper tissues.

Other PTC-RT-related problems are even more elusive and finding ways to overcome them may be difficult, if possible at all.

The availability of a PTC-bearing transcript, which can affect the efficiency of the nonsense suppression, is one such issue. In the cell, mRNA availability is strictly related to the efficiency of the NMD process (Miller & Pearce, 2014). When NMD is efficient, the level of mutant mRNA is noticeably reduced and, even if potent nonsense suppressing drugs are provided, the amount of functional protein is very low (Kuzmiak & Maquat, 2006). In the study using gentamicin as the PTC-RT-stimulating drug in CF patients carrying the same W1282X nonsense mutation, the response to gentamicin was enhanced in the individuals, in whom the level of mutant mRNA was high due to low NMD efficiency (Linde et al., 2007). These results suggest that the level of PTC-bearing transcripts might be a limiting factor in the response to gentamicin treatment. Some reports suggested that even a moderate induction of PTC-RT (e.g. by using AAGs) may promote a stabilization of mutant transcripts and counteract the NMD process (Bedwell et al., 1997). This hypothesis has been later supported by several reports (Floquet et al., 2011; Salvatori et al., 2009; Bellais et al., 2009).

Since the level of mutated transcript expression is of a paramount importance for PTC-RT therapy, blocking NMD by pharmaceutical agents has been considered as the solution to the problem, and a number of studies have explored this path (Usuki et al., 2004; Usuki et al., 2006; Durand et al., 2007; Keeling et al., 2013; Gotham et al., 2016).

Inhibitors of hSMG-1 kinase, which is an essential protein for the regulation of NMD process, form one of the groups of tested compounds (represented by wortmannin and caffeine) (Usuki et al., 2004). In the experiments performed in fibroblasts derived from patients with Ullrich’s disease, caused by PTC in the collagen VI gene, the use of these agents resulted in a correct assembly of collagen VI, despite its truncated C-terminus and formation of a partially functional extracellular matrix (Usuki et al., 2004; Usuki et al., 2006). Another molecule, a small polycyclic indole derivative, NMDI-1, was shown to block the NMD process by interfering with the interaction between key NMD factors, hSMG5 and hUPF1. This led to the stabilization of the hyperphosphorylated form of hUPF1 and its sequestering in cytoplasmic P-bodies (Durand et al., 2007). NMDI-1 was shown to be ~ 1500-fold more effective than caffeine in attenuating NMD; at the same time, it did not have any influence on cell growth and no significant effect on protein synthesis (Keeling et al., 2013). Short term studies in a mouse model of mucopolysaccharidosis (MPS I-H) have shown that a co-administration of NMDI-1 with gentamicin restored 50% more α-L-iduronidase activity than AAG administered alone (Keeling et al., 2013). Chemical synthesis of NMDI-1 is complicated and poorly efficient; however, there were made attempts to synthesize a close NMDI-1 analog, VG1, with similar NMD inhibiting potential and significantly less complicated synthesis protocol (only 6 synthesis steps instead of 13 reported previously) (Keeling et al., 2013; Gotham et al., 2016).

Another promising NMD inhibitor, amlexanox, is a long-used drug with anti-allergic and anti-inflammatory properties (Makino et al., 1987). Amlexanox has been approved by the FDA for the treatment of canker sores, aphthous ulcers and asthma; it is also currently in a phase II clinical trial for the treatment of diabetes (ClinicalTrials.gov; id: NCT01842282). The recent studies showed that amlexanox can inhibit NMD process and promote synthesis of PTC-bearing mRNAs (Gonzalez-Hilarion et al., 2012). The increase in the amount of PTC-bearing mRNA does not affect general translation or expression of potential NMD substrates (Gonzalez-Hilarion et al., 2012). In the cell lines from CF patients, amlexanox alone caused an increase of cAMP-dependent halide efflux, suggesting presence of the functional CFTR protein, probably due to the suppression of PTC in the mutated *CFTR* transcript. The increase in CFTR activity after treatment with amlexanox alone was ~ 3 times higher than after treatment with other PTC-RT inducing compounds, G418 or PTC124. Although the initial results for these agents seem promising, long-term safety and effectivity studies are required before the clinical use of these compounds becomes possible. Efficacy of amlexanox as a readthrough compound and a potent NMD inhibitor was confirmed recently in cells derived from patients with recessive dystrophic epidermolysis bullosa, where a significant increase in a full-length protein level translated from PTC-bearing transcripts was observed (8–80%), compared do the wild-type protein level (Atanasova et al., 2017). The recovered protein was also functional and stable.

The inherent problem associated with the PTC-RT-based therapies is related to the fact that the level of the induced readthrough can vary considerably for different PTC introducing mutations, such that only a subset of patients would be likely to profit from the therapy (Malik et al., 2010; Woodley et al., 2017; Finkel et al., 2013; Nagel-Wolfrum et al., 2016). This variability depends on the stop codon itself and/or on the sequence context of PTC (Dabrowski et al., 2015). The mechanism of the induced stop codon suppression still remains not fully understood, complicating the prediction of PTC-RT efficiency based on the nucleotide context of the PTC mutation. To some extent, this problem may be solved if the efficient pair of the PTC and a specific readthrough compound is found. This however has to be done in an empirical way –to predict which patients are likely to take benefits from the PTC-RT therapy, it is necessary to experimentally determine the PTC-RT level of each nonsense mutation in preclinical settings (Floquet et al., 2012). Moreover, one has to keep in mind that some of the PTCs, in specific sequence context, may be resistant to the drug-induced PTC-RT (Bukowy-Bieryllo et al., 2016).

Even if the full-length protein is produced after drug-induced PTC-RT, the important question about the identity of the amino acid incorporated during decoding of the premature stop still remains. If it is the same amino acid as that present in the normal protein, the question can be disregarded. However, if the amino acid is different from the wild-type, it can lead to synthesizing a potentially non-functional protein, with different biochemical properties or impaired stability. A number of studies showed that, even if the full-length proteins were produced after administration of PTC-RT-inducing drugs, their functional activity and subcellular localization were often impaired (Schulz et al., 2002; Yao et al., 2009; Simon et al., 2010; Brumm et al., 2012).

Some preferences for the amino acid incorporated during the PTC decoding can be predicted from the near-cognate anticodons (Dabrowski et al., 2015). However, this is not the unequivocal indicator. In vitro studies in rabbit reticulocyte lysates identified Trp, Arg, and Cys incorporated at the UGA codon (Feng et al., 1990). Studies addressing the insertion of amino acids at UAA and UAG codons in viral mRNAs revealed only the presence of Gln (Feng et al., 1990; Yoshinaka et al., 1985). Recent readthrough studies using mass-spectrometry shed more light on this issue (Roy et al., 2015; Roy et al., 2016; Xue et al., 2017). Sequence analysis of full-length readthrough products in the yeast system (with reporter vectors containing different stop codons) has shown that Gln, Lys, and Tyr incorporated at UAA and UAG, whereas Trp, Arg, and Cys were inserted at UGA. This was irrespective of whether readthrough is endogenous, or induced. However, the frequency of individual amino acids insertion differed for specific nonsense codons and readthrough compound; Tyr and Gln were incorporated with comparable frequency at UAA (~ 45% and ~ 55%, respectively), Gln dominated at UAG (~ 80%) and Trp at UGA (~ 86%). Interestingly, for all these amino acids, the nc-tRNAs mispaired at position 1 or 3 of nonsense codons. Slightly different results were obtained a human cell line transfected with reporter vectors containing different stop codons (Roy et al., 2016) (Table 1). The frequencies of the incorporated amino acids were different and mispairing at position 2 of nonsense codons was allowed. Nevertheless, the set of possible amino acids incorporated at PTCs was similar to that in the yeast system. The newest evidence indicates that, after stimulation with G418, identity of inserted amino acids depends on the nucleotide context of the same stop codon (UGA). This confirms, that mRNA sequence context plays a key role in near-cognate tRNA selection during PTC-RT (Beznosková et al., 2016; Xue et al., 2017).

**Table 1:** Amino acids inserted during readthrough

Basal PTC-RT							
UGA			UAA		UAG		
Arg (CGA) (58.6 ± 2.3%)	Trp (UGG) (41.4 ± 2.3%)	Cys (UGU/C) -	Tyr (UAU/C) (67.5 ± 8.9%)	Gln (CAA) (32.4 ± 8.9%)	Tyr (UAU/C) (73.1 + 15%)	Gln (CAG) (25.2 ± 13.7%)	Trp (UGG) (1.3 ± 1.4%)
Stimulated PTC-RT PTC124 (30 uM)							
UGA			UAA		UAG		
Arg (CGA) (69.7 ± 11.3%)	Trp (UGG) (28.8 ± 11.4%)	Cys (UGU/C) (0.7 ± 0.7%)	Tyr (UAU/C) (57.9 ± 11.3%)	Gln (CAA) (39.9 ± 11.7%)	Tyr (UGG) (43.9 ± 20.9%)	Gln (CAG) (53.2 ± 20.3%)	Trp (UGG) (1.8 ± 1.9%)
Stimulated PTC-RT G418 (150 uM)							
UGA			UAA		UAG		
Arg (CGA) (64.5 ± 11.8%)	Trp (UGG) (17.9 + 6.8%)	Cys (UGU/C) (17.7 ± 8.0%)	Tyr (UGG) (47.9 ± 14.1%)	Gln (CAA) (52 ± 14.2%)	Tyr (UAU/C) (10.8 ± 7.0%)	Gln (CAG) (86.5 ± 8.3%)	Lys (AAG) (2.0 ± 0.8%)

The frequency of amino acids inserted during either basal or stimulated readthrough (PTC124 or G418) in the human cell line transfected with reported vectors containing different stop codons (based on Roy et al., 2016)

An important issue in PTC-RT based therapies is the minimal amount of the full-length protein that should be restored to achieve the functional and – hopefully – therapeutic effects. In each disease, this level is different: in CF, 30–35% of normal CFTR activity is sufficient; in DMD – 20-30% of the full-length dystrophin (but according to others, even 1% is sufficient); in MPS I-H, even 0.4–1% of the α-L-iduronidase level is enough to alleviate disease symptoms (Hoffman et al., 1988; Keeling et al., 2014). Unfortunately, the effective amount of the full-length protein has to be examined for each gene and disease separately.

## Conclusions

To date, more than 100 studies have shown that the stimulation of PTC-RT may result in the partial restoration of the expression of deficient proteins that underlie ~ 40 different genetic diseases (Lee & Dougherty, 2012; Nagel-Wolfrum et al., 2016). However, no study has demonstrated a remarkable improvement of patients’ health or significant alleviation of the disease phenotype. The establishment of a truly efficient PTC-RT-based therapy requires new, more potent compounds with less toxicity. It can be achieved by the modification of available drugs or by searching for completely new compounds with higher nonsense suppression potential. Additional enhancement might be ensured by co-administration of readthrough compounds with some enhancers, like inhibitors of NMD process or aminoglycoside potentiators, such as CDX5–1. Despite the promising results of the basic research and clinical trials, plenty of work is still needed to better understand/predict ADME (absorption, distribution, metabolism and excretion) characteristics, pharmacokinetics (PK) and pharmacodynamics (PD) of a drug, before these new drugs can be considered as a clinical therapeutic option (Lee & Dougherty, 2012).

## Abbreviations

AAGs: Aminoglycoside antibiotics
eRF1/3: Eukaryotic release factor 1/3
GTTR: Texas Red-tagged gentamicin
N c-tRNA: Near-cognate tRNA
NMD: Nonsense-mediated mRNA decay
NTC: Normal termination codon
PAA: Poly-L-aspartic acid
PABP: Poly(A)-binding proteins
PTC: Premature termination codons
PTC-RT: Premature termination codons readthrough
ROS: Reactive oxygen species
RT: STOP codon readthrough
